# In-home solid fuel use and cardiovascular disease: a cross-sectional analysis of the Shanghai Putuo study

**DOI:** 10.1186/1476-069X-11-18

**Published:** 2012-03-28

**Authors:** Mi-Sun Lee, Jing-qing Hang, Feng-ying Zhang, He-lian Dai, Li Su, David C Christiani

**Affiliations:** 1 Environmental and Occupational Medicine and Epidemiology Program, Department of Environmental Health, Harvard School of Public Health, Boston, MA, USA; 2 Shanghai Putuo District People's Hospital, Shanghai, China; 3 The Massachusetts General Hospital and Harvard Medical School, Boston, MA, USA

**Keywords:** Household fuels, Cardiovascular disease, Indoor air pollution, Chinese

## Abstract

**Background:**

Although recent research evidence suggests an association between household air pollution from solid fuel use, such as coal or biomass, and cardiovascular events such as hypertension, little epidemiologic data are available concerning such exposure effects on cardiovascular endpoints other than hypertension. We explored the association between in-home solid fuel use and self-reported diagnoses of cardiovascular endpoints, such as hypertension, coronary heart disease (CHD), stroke, and diabetes.

**Methods:**

We analyzed 14,068 Chinese adults, aged 18 years and older. Odds ratios (OR) and the corresponding 95% confidence intervals (CI) were estimated using logistic regression models for the risk of each outcome after adjusting for potential confounders.

**Results:**

The use of solid fuel in home was significantly associated with an increased risk for hypertension (OR 1.70, 95% CI 1.40 to 2.07), CHD (OR 2.58, 95% CI 1.53 to 4.32), and diabetes (OR 2.48, 95% CI 1.59 to 3.86), after adjusting for potential confounders. Compared with individuals in the lowest tertile of the duration of solid fuel exposure, those in the highest tertile of the duration of solid fuel exposure had an increased odds of hypertension (OR 1.73, 95% CI 1.45 to 2.06), stroke (OR 1.87, 95% CI 1.03 to 3.38), and diabetes (OR 3.18, 95% CI 2.11 to 4.78).

**Conclusions:**

Our data suggest that in-home solid fuel exposure maybe associated with increased risk for hypertension, CHD, stroke, and diabetes in the Chinese adult population. Further large-scale longitudinal studies are warranted to confirm these findings.

## Background

Indoor air pollution (IAP) from solid fuels, mainly biomass and coal, ranked as one of top ten environmental risk factors of global burden of disease by the World Health Organization [1]. Epidemiologic studies have shown that indoor pollution from the use of solid fuels is associated with acute respiratory infections (ARIs), chronic obstructive pulmonary disease (COPD), and lung cancer [2-5], but limited studies are available on the cardiovascular disease (CVDs), which remains the leading cause of death worldwide. Potential biological mechanisms include oxidative stress, promotion of inflammation with a systemic release of cytokines, and blood coagulation [6].

The combustion of solid fuels in the home release substantial pollutants such as respirable particulate matter (PM), polycyclic aromatic hydrocarbons (PAHs), heavy metals, and many other organic pollutants [7] which have been linked to CVDs. According to recent update to the scientific statement from the American Heart Association (AHA), PM exposure can trigger acute cardiovascular events and accelerate chronic CVDs [8]. In China, CVDs is expected to increase considerably, and the future trends in blood pressure, diabetes, total cholesterol, and body mass index may drive the CVD epidemic during the next 20 years [9].

The present study aimed to examine whether in-home solid fuel exposure from cooking and heating is associated with adverse cardiovascular outcomes, focusing in particular on hypertension, coronary heart disease (CHD), stroke, and diabetes in China. The findings of this study has important public health implications because more than 70% of Chinese homes, mostly is rural areas, still use solid fuels [4], which may lead to continuing potential health problems.

## Methods

### Study design and data collection

The Institutional Review Boards of the Harvard School of Public Health and the Putuo District People's Hospital approved this study. The Shanghai Putuo study, a collaboration between Harvard School of Public Health and Shanghai Putuo District People's Hospital, started to recruit subjects from August 2007 to July 2009 from Shanghai's Putuo District in China. Study subjects were recruited based on random selection from census track data with following eligible criteria: age 18 years and older with no restrictions with regard to prior health history. Of the total 19,620, 14,068 (71.7%) subjects aged ≥ 18 years provided written informed consent to participate in this study with the baseline clinical examination and questionnaire. The participants were recruited without previous knowledge of their disease or exposure status. We did not inform the study participants of the exact aim or hypothesis of the research reported in here.

All participants were interviewed face-to-face in person by trained personnel, using structured questionnaires. All interviewers received at least one day of survey-specific training and performed test interviews before beginning the survey. The questionnaires include socio-demographic factors (age, gender, education, marital status and household income), smoking history (smoking status, pack-years of smoking, and second-hand smoke), occupational history, medical history, and household fuel exposures. Individual's household exposure to solid fuels (coal and biomass) from cooking and heating was assessed in the questions: ever used, duration (the number of years for cooking/heating using solid fuels), total amount (as calculated by multiplying the number of fuel used by the annual amount of fuel used), and lifetime average amount (as calculated by multiplying duration by annual amount by dividing age).

The outcome of interest, stroke, CHD and diabetes mellitus, in current study was assessed by self-reported questionnaire ascertained by a subject answered "Yes" to the question only if it was doctor-diagnosed disease during their lifetime. Blood pressure was measured by physician, based on standardized methods, using a mercury manometer with the subject in a sitting position after 5 min rest. Hypertension was defined as a systolic blood pressure (SBP) ≥ 140 mmHg or a diastolic blood pressure (DBP) ≥ 90 mmHg. Anthropometric measurements such as height, weight, and waist circumference were taken during the physical examination. Body mass index (BMI) was calculated by dividing body weight in kilograms by the square of height in meters. Waist circumference was measured in centimeters at the mid distance between the top of the iliac crest and the bottom of rib cage. Trained personnel performed all measurements.

**Figure 1:**
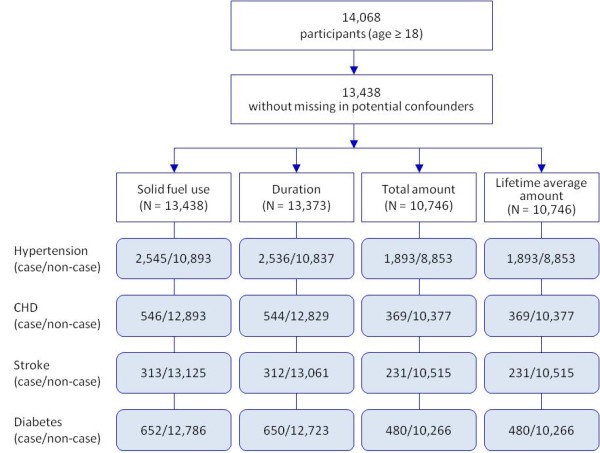
**Flow of study subjects**.

Of the 14,068 participants, 630 with missing potential confounders data including education, BMI, waist circumference, smoking, pack-years of smoking, or second-hand smoke, were excluded; 13,438 (96% of 14,068) were included in the analyses (Figure 1). Of the 13,438 participants, the subjects with missing in duration on solid fuel use (n = 65), total amount and lifetime average amount (n = 2,692) were also excluded. This left 13,373 subjects for the final analysis of duration of solid fuel use and 10,746 subjects for the analysis of total amount and lifetime average amount of solid fuel use, respectively.

### Statistical analysis

Household exposure to solid fuel for cooking and heating was expressed as use of solid fuels (ever users versus nonusers), duration of solid fuel use in years, and total amount of solid fuel use in kilograms, and average amount of solid fuel use in kilograms per year, by assessing on a continuous scale and categorized each into tertiles based on exposure distribution. Logistic regressions were applied to test the association between household solid fuel exposure and each CVD outcomes after adjusting for covariates. The core covariates considered in the models were age (years), gender, education level (less than high school, high school, and above college or more), smoking status (current, former, and never smoker), pack-years of smoking, second-hand smoke (ever and never), waist circumference [≥ 80(female) or 90(male), < 80(female) or 90(male)], and BMI ( < 18.5, 18.5-24.9, and ≥ 25.0). Such models have the form:

$$\frac{\text{In}\left(P\left(y=1|x_{1},...,x_{p}\right)\right)}{\left(1-P\left(y=1|x_{1},...,x_{p}\right)\right)}=\beta_{0}+\beta_{1}x_{1}+\beta_{2}x_{2}+\ldots +\beta_{p}x_{p}+e.$$

where y is the each CVD outcomes and *x_p _*represents the values of the explanatory variables. Trend tests were conducted by treating the exposure variables categorized into tertiles and putting into the models as a continuous variable, to analyze whether risks changed with the tertile scale of duration and amount of solid fuel exposure. We also tested the association of solid fuel use with each CVD end point for sub-population stratified by age ( < 40 and ≥ 40) as CVD is a leading cause of death in Chinese above 40 years [10], gender (male and female) and smoking status (ever and never). Due to the small number of former smokers, smoking status was stratified as ever (current plus former smoker) and never smokers. All statistical analyses were performed using SAS version 9.2 (SAS Institute Inc., Carry, NC, USA).

## Results

### Characteristics of the study participants

Table 1 presents demographic and clinical characteristics of the study population. The study population consisted of 6,463 men (45.9%) and 7,605 women (54.1%) with a mean age of 48.6. Overall, 2,673 (19.0%) were hypertensive and 572 (4.1%), 339 (2.4%), and 684 (4.9%) had CHD, stroke and diabetes mellitus, respectively. Table 2 shows the biological and potential cardiovascular risk factors, stratified by household solid fuel use. After adjusting for all potential confounders, statistical significant differences between ever users and nonusers were found in age, gender, education, second-hand smoke, and smoking status.

**Table 1:** General characteristics of the study population, N (%) or Mean ± SD

Variables	Overall (N = 14,068)
Age, yrs	48.6 ± 16.6
18-29	2,323(16.5)
30-39	2,211 (15.7)
40-49	2,238 (15.9)
50-59	3,813 (27.1)
60 ≥	3,483 (24.8)
Gender	
Male	6,463 (45.9)
Female	7,605 (54.1)
Education	
Less than high school	5,661 (40.3)
High school	6,604 (43.1)
College or above	2,341 (16.6)
BMI^a^, kg/cm^2^	23.1 ± 3.2
≥ 25.0	3,599 (25.6)
18.5-24.9	9,525 (67.9)
< 18.50	905 (6.5)
Waist circumference^b^, cm	
≥ 80 (female) or 90 (male)	10,127 (72.5)
< 80 (female) or 90 (male)	3,845 (27.5)
Second-hand smoke	
Never	3,106 (22.1)
Ever	10,960 (77.9)
Smoking	
Current smoker	3,215 (23.5)
Ex-smoker	651 (4.8)
Never smoker	9,791 (71.7)
Amount of smoking, pack-years	6.8 ± 21.3
Household solid fuel exposures	
Duration, yr	19.0 ± 1.54
Total amount, 1000 kg	12.9 ± 12.6
Lifetime average amount, kg/year	236.3 ± 209.6
Hypertension	
Yes	2,673 (19.0)
No	11,395 (81.0)
Coronary heart disease	
Yes	572 (4.1)
No	13,496 (95.9)
Stroke	
Yes	339 (2.4)
No	13,729 (97.6)
Diabetes	
Yes	684 (4.9)
No	13,384 (95.1)

*Note*: The number of subjects may not total 100% (N = 14,068) due to missing.

^a ^Classified by the international classification of adult underweight ( < 18.5), normal (18.5-24.9), and overweight or obese (≥ 25.0) according to WHO cut-off point; ^b ^Classified by the Asia-Pacific guidelines for the management of obesity (WHO 2000).

**Table 2:** General characteristics of the study population, stratified by in-home solid fuel use, N (%) or Mean ± SD

Variables	Ever user (n = 11,013)	Nonuser (n = 3,055)	*P*-value^c^
Age, yrs	52.9 ± 15.0	33.2 ± 12.5	< 0.001
18-29	842 (36.2)	1,481 (63.8)	
30-39	1,363 (61.7)	848 (38.3)	
40-49	1,898 (84.8)	340 (15.2)	
50-59	3,558 (93.3)	255 (6.7)	
60 ≥	3,352 (96.2)	131 (3.8)	
Gender			
Male	5,053 (78.2)	1,410 (21.8)	0.01
Female	5,960 (78.4)	1,645 (21.6)	Reference
Education			
Less than high school	5,248 (92.7)	413 (7.3)	< 0.001
High school	4,502 (74.2)	1,562 (25.8)	0.76
College or above	1,262 (53.9)	1,079 (46.1)	Reference
BMI^a^, kg/cm^2^			
≥ 25.0	3,063 (85.1)	536 (14.9)	0.10
18.5-24.9	7,342 (77.1)	2,183 (22.9)	0.76
< 18.5	577 (63.8)	328 (36.2)	Reference
Waist circumference^b^, cm			
≥ 80 (female) or 90 (male)	8,280 (81.8)	1,847 (18.2)	0.06
< 80 (female) or 90 (male)	2,665 (69.1)	1,180 (31.9)	Reference
Second-hand smoke			
Never	2,149 (69.2)	957 (30.8)	< 0.001
Ever	8,862 (80.9)	2,098 (19.1)	Reference
Smoking			
Current smoker	2,717 (84.5)	498 (15.5)	0.01
Ex-smoker	604 (92.8)	47 (7.2)	0.78
Never-smoker	7,383 (75.4)	2,408 (24.6)	Reference
Amount of smoking, pack-years	8.0 ± 23.1	2.4 ± 11.2	0.24

*Note*: The number of subjects may not total 100% (N = 14,068) due to missing.

^a ^Classified by the international classification of adult underweight ( < 18.5), normal (18.5-24.9), and overweight or obese (≥ 25.0) according to WHO cut-off point; ^b ^Classified by the Asia-Pacific guidelines for the management of obesity (WHO 2000); ^c ^*P*-value for comparing the difference between ever users versus nonusers, given by logistic regression analysis after adjusting for all potential confounding variables.

### Association of household solid fuel use with CVD outcomes

The associations of solid fuel use for cooking or heating, as assessed by ever use, duration, total amount, and lifetime average amount throughout the lifetime, with hypertension, CHD, stroke, and diabetes are given in Table 3. We controlled for biological susceptibility factors [11], namely age and gender (Model 1), and then additionally controlled for potential cardiovascular risk and extrinsic susceptibility factors including education, smoking, pack-years, SHS, BMI, and waist circumference (Model 2). Solid fuel use was associated with an increased risk for hypertension (OR 1.70, 95% CI 1.40 to 2.07), CHD (OR 2.58, 95% CI 1.53 to 4.32) and diabetes (OR 2.48, 95% CI 1.59 to 3.86) after accounting for potential cardiovascular risk and confounding factors (Model 2). Longer duration of solid fuel use were associated with an increased odds of hypertension (OR 1.73, 95% CI 1.45 to 2.06), stroke (OR 1.87, 95% CI 1.03 to 3.38), and diabetes (OR 3.18, 95% CI 2.11 to 4.78), in the highest compared with lowest tertile. Greater amount of solid fuel use throughout the lifetime were associated with an increased odds of hypertension (OR 1.62, 95% CI 1.34 to 1.97) and diabetes (OR 2.64, 95% CI 1.71 to 4.08), in the highest compared with lowest tertile. These estimates of these effects were not considerably changeable when compared with age- and gender-adjusted model (Model 1).

**Table 3:** Odds Ratio (95% CI) for CVD associated with household solid fuel use

	Hypertension		CHD		Stroke		Diabetes	
	Model 1^†^	Model 2^‡^	Model 1^†^	Model 2^‡^	Model 1^†^	Model 2^‡^	Model 1^†^	Model 2^‡^
Solid fuel use								
Case/non-case	2,545/10,893		546/12,892		313/13,125		652/12,786	
Ever	1.91 (1.58 to 2.30)	1.70 (1.40 to 2.07)	2.56 (1.53 to 4.28)	2.58 (1.53 to 4.32)	1.72 (0.87 to 3.40)	1.60 (0.80 to 3.21)	2.72 (1.75 to 4.23)	2.48 (1.59 to 3.86)
Never	Reference	Reference	Reference	Reference	Reference	Reference	Reference	Reference
Duration (yr, tertile)								
Case/non-case	2,536/10,837		544/12,829		312/13,061		650/12,723	
> 25	1.98 (1.67 to 2.35)	1.73 (1.45 to 2.06)	1.48 (1.01 to 2.16)	1.46 (0.99 to 2.15)	2.02 (1.13 to 3.61)	1.87 (1.03 to 3.38)	3.52 (2.35 to 5.28)	3.18 (2.11 to 4.78)
10-25	1.69 (1.43 to 2.00)	1.51 (1.27 to 1.79)	1.96 (1.34 to 2.85)	1.93 (1.32 to 2.82)	1.51 (0.81 to 2.80)	1.45 (0.78 to 2.71)	3.05 (2.04 to 4.56)	2.83 (1.88 to 4.24)
< 10	Reference	Reference	Reference	Reference	Reference	Reference	Reference	Reference
*P *for trend	< 0.001	< 0.001	0.698	0.727	0.006	0.017	< 0.001	< 0.001
Total amount (1000 × kg, tertile)								
Case/non-case	1,893/8,853		369/10,377		231/10,515		480/10,266	
> 16.72	1.79 (1.49 to 2.16)	1.62 (1.34 to 1.97)	1.27 (0.81 to 1.99)	1.29 (0.82 to 2.04)	1.71 (0.95 to 3.10)	1.62 (0.89 to 2.96)	2.87 (1.86 to 4.41)	2.64 (1.71 to 4.08)
4-16.72	1.65 (1.37 to 1.98)	1.49 (1.23 to 1.80)	2.36 (1.52 to 3.66)	2.39 (1.53 to 3.73)	1.19 (0.63 to 2.24)	1.13 (0.60 to 2.15)	2.97 (1.93 to 4.56)	2.78 (1.80 to 4.26)
< 4	Reference	Reference	Reference	Reference	Reference	Reference	Reference	Reference
*P *for trend	< 0.001	< 0.001	0.103	0.125	0.014	0.023	< 0.001	0.001
Lifetime average amount, kg/year								
Case/non-case	1,893/8,853		369/10,377		231/10,515		480/10,266	
> 340	1.52 (1.29 to 1.78)	1.43 (1.21 to 1.68)	0.77 (0.55 to 1.09)	0.78 (0.55 to 1.10)	1.38 (0.89 to 2.14)	1.34 (0.86 to 2.10)	1.84 (1.33 to 2.54)	1.74 (1.25 to 2.41)
81.8-340	1.35 (1.15 to 1.59)	1.28 (1.08 to 1.50)	1.38 (1.00 to 1.91)	1.38 (0.99 to 1.90)	0.96 (0.60 to 1.53)	0.95 (0.59 to 1.52)	1.99 (1.44 to 2.75)	1.92 (1.38 to 2.65)
< 81.8	Reference	Reference	Reference	Reference	Reference	Reference	Reference	Reference
*P *for trend	< 0.001	< 0.001	0.004	0.006	0.030	0.044	0.006	0.016

^† ^Adjusted for age and gender.

^‡ ^Adjusted for age, gender, education level (less than high school, high school, above college or more), smoking (current, former, never), SHS (ever and never), pack-years, BMI ( < 18.5, 18.5-24.9, ≥ 25.0), and waist circumference.

By age group, similar associations were seen between solid fuel use and the risk of hypertension (OR 1.34, 95% CI 1.09 to 1.66), CHD (OR 1.86, 95% CI 1.11 to 3.12), and diabetes (OR 2.01, 95% CI 1.25 to 3.21) among the group of age ≥ 40 (Table 4). In contrast, little association was seen among subjects with age < 40, although the number of cases with CHD and stroke in this age group was few to none. The interaction term in the model was not statistically significant. By gender, the associations between solid fuel use with CVDs end points were similar for men and women, but the odds of solid fuel use with hypertension was greater among women (OR 2.00, 95% CI 1.49 to 2.71) than men (OR 1.54, 95% CI 1.20 to 1.99) and the difference was statistically significant (*P *for interaction = 0.02). We saw no significant differences by gender in the association of solid fuel use with other CVDs end points. By smoking status, slightly higher ORs for hypertension and CHD were observed among never smokers compared to those among ever smokers and an interaction term in the logistic model was significant, respectively (*P *for interaction = 0.02).

**Table 4:** Odds Ratio (95% CI) ^† ^for CVD associated with household solid fuel use, stratified by age, gender and smoking status

	Hypertension	CHD	Stroke	Diabetes
	OR (95% CI)	OR (95% CI)	OR (95% CI)	OR (95% CI)
Age^a^				
< 40	1.24 (0.71 to 2.18)	NC^d^	NC^d^	0.64 (0.14 to 2.98)
≥ 40	1.34 (1.09 to 1.66)	1.86 (1.11 to 3.12)	1.42 (0.71 to 2.84)	2.01 (1.25 to 3.21)
*P *for interaction	0.34	0.95	0.99	0.44
Gender^b^				
Male	1.54 (1.20 to 1.99)	1.99 (0.95 to 4.19)	1.70 (0.66 to 4.35)	2.41 (1.31 to 4.42)
Female	2.00 (1.48 to 2.71)	3.15 (1.53 to 6.51)	1.48 (0.53 to 4.14)	2.53 (1.31 to 4.86)
*P *for interaction	0.02	0.32	0.97	0.52
Smoking status^c^				
Ever (former and current)	1.39 (0.99 to 1.94)	1.10 (0.49 to 2.47)	1.11 (0.38 to 3.27)	3.16 (1.27 to 7.87)
Never	1.84 (1.45 to 2.33)	3.65 (1.85 to 7.22)	1.96 (0.78 to 4.89)	2.21 (1.32 to 3.68)
*P *for interaction	0.02	0.02	0.51	0.72

^a ^Adjusted for age, gender, education level (less than high school, high school, above college or more), smoking (current, former, never), SHS (ever and never), pack-years, BMI ( < 18.5, 18.5-24.9, ≥ 25.0), and waist circumference.

^b ^Adjusted for age, education level (less than high school, high school, above college or more), smoking (current, former, never), SHS (ever and never), pack-years, BMI ( < 18.5, 18.5-24.9, ≥ 25.0), and waist circumference.

^c ^Adjusted for age, gender, education level (less than high school, high school, above college or more), SHS (ever and never), pack-years, BMI ( < 18.5, 18.5-24.9, ≥ 25.0), and waist circumference.

^d^ORs and 95%CIs are not estimated due to small number of cases [number of cases for CHD (n = 8) and for stroke (n = 0), respectively].

## Discussion

In this large-scale population-based cross-sectional study, we found that solid-fuel user, particularly when exposed to cooking and heating, experienced higher risk of hypertension, coronary heart disease and diabetes, compared to non-solid fuel users, especially among the general population 40 years of age and older in China. There was a trend towards increased risk for hypertension, stroke, and diabetes for longer duration and greater amount of in-home solid fuel exposure throughout lifetime.

Epidemiologic data on the cardiovascular effects of solid fuel use in home are very limited. Though lack of epidemiologic data makes direct comparison with our results difficult, numerous cardiovascular epidemiologic studies showed associations with indoor air pollutants such as PM, PAHs, and heavy metals [7,12,13], which contribute to indoor high particulate level from solid fuel emissions [14]. A 10 μg/m^3 ^increase in total suspended particle (TSP) generated from burning sugar cane plantations led to a 12.5% increase in hypertension-related hospital admissions in São Paulo State, Brazil [15]. In Australia, exposure to particles with a mean aerodynamic diameter less than 10 μm (PM_10_) derived from ambient biomass smoke was associated with an elevated risk of ischemic heart disease, whereas no association was observed with cardiovascular admissions in total [16]. Indian women who cooked with biomass fuel had higher prevalence of hypertension than women who performing the cooking with cleaner fuel LPG [17]. Chronic exposure to fine particulate matter (PM_2.5_) from household use of open fires was associated with an elevated blood pressure among Guatemalan women [18]. More recent epidemiologic data showed that personal exposure to PM_2.5 _from biomass combustion was positively associated with systolic and diastolic blood pressure (BP) in women in rural China [19].

Epidemiologic studies regarding cardiovascular effects of solid fuel use have conducted mainly among women and found positive associations with hypertension and blood pressure [17-19], as our finding is consistent. For hypertension, we observed stronger association among women, with an OR of 2.00 for women and 1.54 for men, but the associations of solid fuel use with other outcomes were inconsistent by gender, as previous studies for air pollution related cardiovascular effects by gender are not consistent. It is assumed that women are more at risk for in-home solid fuel exposure because they generally spend more time in the home and do more of the cooking [20], but lack of information on amount of time spent of the cooking did not allow us to resolve it. However, we found the association with an increased risk of hypertension in both genders, suggesting household solid fuel use may also be an important risk factor for men. Our analysis stratified by smoking showed that the association for household solid fuel use and hypertension and CHD was stronger among never smokers and small or null among ever smokers. Epidemiologic studies suggest that a stronger environmental pollutants-CVD association is seen among never smokers compared with current smokers suggested that this may indicate "pressure" effects [21]. Our finding stratified by cigarette smoking indicates that residual confounding by smoking effects does not seem to explain our findings, but we cannot completely rule out the fact that it may partly mask it.

The mechanisms involved in the potential toxic effects of in-home solid fuel exposure-related cardiovascular system are unclear, but the main mechanisms may be linked to inflammation through the generation of reactive oxygen species (ROS) and oxidative stress. An experimental study showed that increased oxidative stress is responsible for activation of apoptosis in cardiac cells in the heart [22]. A recent study in China showed that the use of biomass for cooking greatly elevates PM exposures, particularly for those performing the cooking [14]. These PM contains pro-oxidative organic hydrocarbons, such as PAHs [7], particularly in particle phase, that may cause oxidative DNA damage and secretion of pro-inflammatory cytokines and chemokines that can lead to cardiovascular effects [6,13]. PAH exposure from the cooking is associated with oxidative DNA damage, assessed by 8-hydroxy-2'-deoxyguanosine (8-OHdG), among Chinese restaurant workers [23]. Chronic exposure to biomass smoke increased the number of leukocyte-platelet aggregates among Indian women [24], and is considered a risk factor for thrombotic disease such as myocardial infarction [25], stroke [26], and unstable angina [27].

We acknowledge several limitations to our study. First, given the cross-sectional design, reverse causation may be possible if an individual with hypertension, CHD, stroke, or diabetes is more likely to use a solid fuel for cooking or heating. However, although this is unlikely, further prospective investigation is warranted. Second, the exposure assessment of solid fuel was based on questionnaire data, not on observed measurements, and therefore might not represent an individual's actual exposure. Third, possible selection bias due to lack of data on lifetime average amount of solid fuel use (24% of 14,068) may raise concern. To check this, we analyzed the association between each CVD outcome and missing in lifetime average amount of solid fuel use among ever users. No association was found with CHD, stroke, and diabetes among the subjects with missing data in lifetime average amount of solid fuel use compared with the subjects without missing, whereas an increased risk of hypertension was found. Our findings are consistent when we considered various household solid fuel exposure indexes such as use of solid fuel, duration, total amount, and lifetime average amount. Therefore, it is unlikely that our observed findings are due to possible selection bias by lack of data in lifetime average amount of solid fuel use. Although qualitative assessment used in our study could suggest the source of exposure in this large population, we cannot provide information on which constituents of in-home fuel exposure had detrimental cardiovascular effects. Further longitudinal research using biomarkers of exposure are needed to investigate the specific chemicals of in-home solid fuel emissions and therefore better understand cardiovascular impact of in-home fuel exposure.

## Conclusions

Our large-scale population-based study suggests that the use of solid fuel in the home maybe associated with an increased risk of hypertension, CHD, and diabetes in the general urban Chinese adult population. The longer the exposures to solid fuel in home, the greater their risk for cardiovascular events, implying evidence of chronic toxicity of solid fuel use in home environment. The results of this study, however, need be interpreted with caution due to the cross-sectional nature of the design. Additional longitudinal study is needed to confirm our findings.

## Abbreviations

BMI: body mass index; CI: confidence interval; CHD: coronary heart disease; CVD: cardiovascular disease; DBP: diastolic blood pressure; OR: odds ratio; PM: particulate matter; PM_10_: particles with a mean aerodynamic diameter less than 10 micrometers; PM_2.5_: particles with a mean aerodynamic diameter less than 2.5 micrometers; PAHs: polycyclic aromatic hydrocarbons; ROS: reactive oxygen species; SBP: systolic blood pressure; SHS: second-hand smoke; TSP: total suspended particle.

## Competing interests

The authors declare that they have no competing interests.

## Authors' contributions

MSL and JQH participated in study design, data collection, statistical analysis and interpretation of the results, and drafted the manuscript. DCC originated the study concept, raised funds for the study, participated in study design, statistical analyses, and critically revised the manuscript. FYZ, HLD, and LS collected data, analysis, and interpretation of data. All authors read and approved the final manuscript.
